# The Disinfection of Rooms

**Published:** 1902-04-05

**Authors:** 


### THE DISINFECTION OF ROOMS.

The Medical Officer of Health for London (Mr. Shirley Murphy) has recently presented a report on the subject of disinfection, containing a joint report by Drs. Klein, Houston and Gordon on a series of experiments which they had made in regard to the comparative efficiency of a series of disinfecting substances under the ordinary conditions of their use in dwelling houses. A room in an unoccupied house in the possession of the Council was in the first instance utilised for the purpose of testing methods of gaseous disinfection, and later a room of about the same size in the shelter of the Finsbury Borough Council. Among fluid disinfectants carbolic acid, permanganate of soda, bleaching powder and corrosive sublimate were tested; while among gaseous disinfectants formalin and sulphurous acid gas were made the subject of experiment, the disinfections in this branch of the inquiry being supervised by Dr. Newman, Medical Officer of Health for Finsbury. Four different sorts of material which in ordinary practice often require to be disinfected, namely, cloth, unvarnished or unpainted wood, linen, and wall-paper, were chosen for experiment, and to them microbes distributed in broth, in milk, or in melted gelatine, were liberally applied, and the charged materials were then exposed to the action of the various disinfectants. The results were interesting, tending, so far as they go, to shake our faith in gaseous and in certain much-used fluid disinfectants, while showing on the other hand that a solution of corrosive sublimate, of the strength of 1 in 1,000, is to be trusted in all cases. As a summary of the results obtained it may be stated that the typhoid bacillus was killed by all the disinfectants except Condy's fluid and bleaching powder. The bacillus diphtheriæ was killed by formalin and sulphur dioxide. The vibrio of cholera was in each experiment destroyed by all the disinfectants except Condy's and bleaching powder. Condy's fluid was practically of negative value, and bleaching powder was not always efficacious on one hour's exposure, but was successful with 24 hours. Bacillus pyocyaneus was acted on in much the same way, being killed in each experiment by all except Condy's fluid and bleaching powder. Anthrax spores were only destroyed with certainty by perchloride of mercury, the other disinfectants almost invariably failing when wood and cloth were the materials experimented on. For tubercle bacilli carbolic acid and perchloride of mercury were the only disinfectants efficacious on each occasion. Neither formalin nor sulphur dioxide were efficacious for the wood or cloth infected with this bacillus. It is worthy of note that in regard to the gaseous disinfectants a great difference was observed between their action on infected wood and cloth, on the one hand, and upon linen and paper on the other, the disinfection being much less complete in the cases in which the infecting solutions had soaked deeply into the materials used, as in the case of wood and cloth, than in those where the infection was more limited to the surface.

The experiments detailed certainly seem to drive one to actual scrubbing of walls as the only reliable method of purification. If this be so the Public Health Department of the County Council would, we think, do well to carry their experiments a little further and ascertain how far perchloride of mercury is to be trusted after walls have been treated with the various alkaline solutions used by decorators for removing papers and for washing down distemper. Our own impression is that when a room has been stripped and scraped, by aid of the solutions commonly employed for the purpose, there is not very much fear of active infection remaining. If, however, we are to employ a "disinfectant" in addition it would be well to know which is the best to use under these circumstances.

